# Are cis-spliced fusion proteins pathological in more aggressive luminal breast cancer?

**DOI:** 10.18632/oncotarget.28438

**Published:** 2023-06-12

**Authors:** Chia-Chia Liu, Xiao-Song Wang

**Keywords:** luminal B breast cancer, RAD51AP1-DYRK4, MEK inhibitor, chimerical transcript

A vast majority of breast cancers (~70%) are estrogen receptor-alpha positive (ER+), for which endocrine therapy is the common treatment. However, recurrence often occurs leading to tumor progression, metastasis, and eventually patient death, and the underlying molecular mechanisms remain poorly understood. Recurrent gene fusions are hallmarks of some cancers that resulted either from chromosomal rearrangements or from cis- or trans-splicing [1–3]. Importantly, selected oncogenic fusions have been matched with effective targeted therapy in several solid tumors. For instance, EML4-ALK, one of the most important oncogenic driver genes of non-small cell lung cancer (NSCLC) uncovered in recent years [4]. In addition to gene fusions resulting from genomic rearrangements, a read-through SLC45A3-ELK4 fusion transcript has been identified in prostate cancer which is associated with disease progression and metastasis [5].

Although the whole genome and RNA sequencing provide an effective way to detect fusion genes, the downstream identification and validation of fusion genes or their products in solid tumor remains a major challenge. We utilize the combination of bioinformatic approach and experimental validation to uncover recurrent fusion genes in different subtypes of breast cancer as exemplified by ESR*1-CCDC170*, the first identified recurrent gene fusion in ER+ breast cancer tumor with more aggressive phenotypes via genetic rearrangement [6, 7] and *BCL2L14-ETV6*, the first reported TNBC-specific recurrent gene fusion associated with cell motility, invasiveness, EMT and chemoresistance [8]. Recently non-genomic chimeric transcripts have been reported in some solid tumors and studied as a pathogenic driver associated with disease progression [2, 5, 9–12]. Through analysis of RNA-seq data from TCGA, we recently identified a neoplastic fusion transcript RAD51AP1-DYRK4 in luminal B breast cancer (~17.5%) showing higher ki67 expression which is an indication of aggressive clinical characteristics. To determine the expression of *RAD51AP1-DYRK4* in normal tissues, among 23 types of normal human tissues including somatic, germ, and fetal tissues, the *RAD51AP1-DYRK4* was only abundantly expressed in testis but not in any of the other tissues, suggesting the importance of this cancer-testis specific chimera in breast cancer. In addition, we evaluated both *RAD51AP1-DYRK4* and *ESR1-CCDC17* in ER+ tumors and found that they are mutually exclusive, suggesting them as independent pathologic events in ER+ breast cancer. The fusion protein is composed of c-terminal truncated RAD51AP1 protein without the interaction domain of RAD51, and it is fused to the out-of-frame DYRK4 peptide resulting in cytoplasmic localization which implies RAD51AP1-DYRK4 exhibits a distinctive biological function from wild-type *RAD51AP1* protein that plays a major role in DNA repair. Another fusion partner, DYRK4 belongs to the conserved family of serine/threonine kinase. However, this gene doesn’t contribute to any in-frame protein sequences to the fusion product. Therefore, it is highly unlikely to act in any role via its kinase domain to the fusion protein. After characterizing the protein product encoded by RAD51AP1-DYRK4, ectopic expression of *RAD51AP1-DYRK4* in T47D cells was established which leads to the activation of MEK/ERK signaling and confers enhanced cell motility and trans-endothelial migration. More importantly, our data revealed that following knockdown of endogenous RAD51AP1-DYRK4 protein overexpressed in MDAMB361 by fusion-specific siRNA, the cell proliferation was significantly inhibited which was not observed in the fusion-negative cell lines. Targeting fusion protein by small molecule inhibitors is a critical but challenging topic, and evaluation of the therapeutic importance is necessary for clinical translation of new discoveries. In this study, we examined the utility of MEK inhibitor trametinib (Mekinist) currently used for treating melanoma with BRAF mutations, in blocking the MEK-ERK signaling driven by *RAD51AP1-DYRK4* fusion. Interestingly, the cells expressing RAD51AP1-DYRK4 protein were remarkably sensitive to MEK treatment and this effect was dependent on the fusion expression, suggesting that RAD51AP1-DYRK4 may endow sensitivity to MEK inhibition in luminal B breast cancer [13]. To our knowledge, this is one of the few non-tradition fusions generated by read-through event in the absence of DNA rearrangement that play an important role in tumorigenesis. Our findings may provide a useful therapeutic approach for treating breast cancer patients who may suffer from early relapse and intrinsic resistance. Advances in the identification of both classic genomic rearrangement and non-genomic fusion events resulting from cis- and trans-splicing events will offer new insights into the pathogenetic mechanism of tumorigenesis, and pave the way to individualized precision medicine for patients. Further studies are needed to understand underlying the mechanisms driven by RAD51AP1-DYRK4 to activate MEK/ERK signaling and elucidate the therapeutic relevance of this discovery in the clinical setting.
